# Spectrum–Effect Relationships as an Effective Approach for Quality Control of Natural Products: A Review

**DOI:** 10.3390/molecules28207011

**Published:** 2023-10-10

**Authors:** Peiyu He, Chunling Zhang, Yaosong Yang, Shuang Tang, Xixian Liu, Jin Yong, Teng Peng

**Affiliations:** School of Pharmacy, Chengdu University of Traditional Chinese Medicine, Chengdu 611137, China; hepeiyu1995@163.com (P.H.); zhangclcdutcm@126.com (C.Z.); ikf0994898@126.com (Y.Y.); tangshuang@stu.cdutcm.edu.cn (S.T.); 15308373463@163.com (X.L.); yongjin@stu.cdutcm.edu.cn (J.Y.)

**Keywords:** spectrum–effect relationships, fingerprints, efficacy, chemometric methods, natural products, traditional Chinese medicine

## Abstract

As natural products with biological activity, the quality of traditional Chinese medicines (TCM) is the key to their clinical application. Fingerprints based on the types and contents of chemical components in TCM are an internationally recognized quality evaluation method but ignore the correlation between chemical components and efficacy. Through chemometric methods, the fingerprints represented by the chemical components of TCM were correlated with its pharmacodynamic activity results to obtain the spectrum–effect relationships of TCM, which can reveal the pharmacodynamic components information related to the pharmacodynamic activity and solve the limitations of segmentation of chemical components and pharmacodynamic research in TCM. In the 20th anniversary of the proposed spectrum–effect relationships, this paper reviews its research progress in the field of TCM, including the establishment of fingerprints, pharmacodynamic evaluation methods, chemometric methods and their practical applications in the field of TCM. Furthermore, the new strategy of spectrum–effect relationships research in recent years was also discussed, and the application prospects of this technology were discussed.

## 1. Introduction

In Asia and other regions, the bioactive compounds in natural products represented by traditional Chinese medicine (TCM) have a wide range of biological activities. Based on the theory and the practice of TCM in the past dynasties, TCM has achieved well-performed therapeutic efficacy in the clinical treatment of diseases, which makes it widely concerned in the world. With the development of modernization and internationalization of the TCM industry, the research on the TCM quality control model has gradually developed from simple observation of appearance traits to microscopic identification and then to specific thin-layer identification and quantitative detection of intrinsic components [1]. However, whether it is a single TCM or a compound preparation, its therapeutic effect is the result of multi-component, multi-target and multi-path cooperation, and the qualitative and quantitative detection of one or several chemical components is difficult to realize in the quality control of TCM. At present, TCM fingerprint technology is widely used in the world as an effective method to evaluate the quality of TCM and ensure its consistency and stability, but its correlation with the efficacy of TCM remains unclear [2]. Some bioaction-guided separation methods and online screening techniques have been effective in the search for bioactive compounds, but the heavy workload and strict experimental equipment limit their high-throughput application in TCM, which makes it more difficult to use them as a general quality control method for TCM [3,4,5,6].

In order to establish the TCM quality evaluation model on the basis of the combination of TCM material basis and efficacy, in 2002, Li et al. proposed to establish the “Spectrum–effect relationships” of TCM by associating the changes of chemical components in the characteristic peak fingerprints with efficacy [7]. The research models of spectrum–effect relationships are as follows: on the basis of samples with differences, the chemical fingerprints of the samples were established, and the pharmacodynamic model and appropriate pharmacodynamic indicators were established. The chemical information and pharmacodynamic data obtained to the maximum extent were correlated and analyzed through data processing methods so as to reveal the pharmacodynamic material basis that could realize the quality control of TCM. Through this research model it cannot only make up for the shortcomings of the traditional quality evaluation model, which only focuses on compounds but ignores the efficacy and realizes the organic combination of fingerprints and pharmacodynamic research to enhance the consistency between fingerprints and efficacy, thus achieving the purpose of quality evaluation of TCM according to the spectrum–effect relationships.

**Figure 1:**
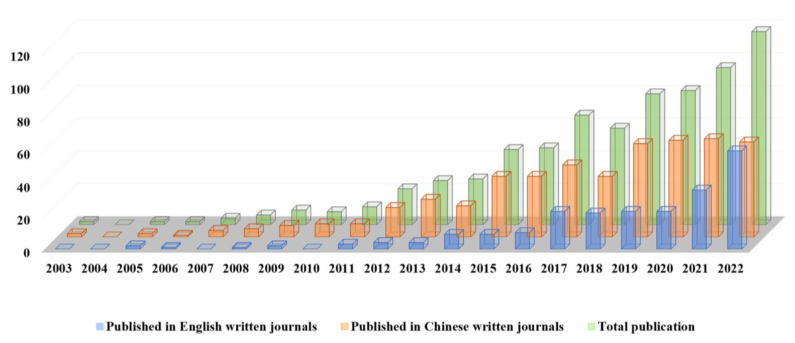
Trends in the number of published research articles on spectrum–effect relationships between 2002 and 2022.

Since the pharmacological information of the active ingredients in the complex matrix of natural products such as TCM can be accurately reflected by the spectrum–effect relationships, in the past 20 years, the study of spectrum–effect relationships has been widely used in the quality evaluation of TCM, the optimization of pharmaceutical process, and the exploration of drug-containing serum. By searching the papers related to spectrum–effect relationships published in Ph.D. and M.Sc. dissertations, online scientific databases including PubMed, SciFinder, ScienceDirect, Scopus, Web of Science, Google Scholar, China National Knowledge Infrastructure, and other databases in the past 20 years, a total of 716 relevant research papers were retrieved after eliminating duplicate articles. These included 230 articles published in English journals and 486 in Chinese journals. The number of published articles showed an increasing trend year by year (Figure 1).

In the early years, some researchers reviewed the research on the spectrum–effect relationships and explained the research objects and data processing methods [8,9,10], but they mainly focused on the elaboration of concepts and lacked practical guidance for the application part, which may not be enough for researchers to fully understand how to complete the experimental design of spectrum–effect relationships research. Especially in recent years, with the development of instruments and the maturity of research systems, researchers have adopted more advanced analytical techniques and pharmacological models to conduct more in-depth studies on the spectrum–effect relationships. Moreover, some novel strategies have also been applied to the research of spectrum–effect relationships, such as efficacy verification by monomeric compound and component knock-out, mechanism exploration based on network pharmacology and molecular docking, pharmacodynamic prediction based on chemometric methods, and evaluating the quality of multi-components based on quantitative analysis of multi-components by a single marker (QAMS). Based on the process of establishing the spectrum–effect relationships model, this review introduces and summarizes in detail the establishment of fingerprints, including the selection of different samples, the selection of appropriate analysis techniques, and the establishment of pharmacological models, including the selection of different animal models, and the selection of data processing methods based on stoichiometric methods. The latest application of the study of the spectrum–effect relationships in the quality evaluation, pharmaceutical process optimization, and exploration of drug-containing serum of TCM was reviewed from a concrete and practical point of view. Furthermore, the cutting-edge strategies applied in spectrum–effect relationships are summarized in this review, and the current challenges and future trends of spectrum–effect relationships research are further discussed. At the same time, this paper attempts to provide readers with the research guide of this research model and provide a reference for the further application of spectrum–effect relationship research in the field of TCM quality control.

## 2. The Establishment Process of Spectrum–Effect Relationships

The chemical compositions of TCM may vary greatly due to plant species, harvesting time, cultivating regions, storage conditions, processing methods and other factors, thus affecting the consistency of its quality. As a comprehensive and quantifiable identification method based on the chemical composition information of TCM, fingerprints have become internationally recognized as the most effective method to control the quality of natural medicine, which can match the characteristics of TCM well and achieve the quality control and analysis of it. After the establishment of the fingerprints, how to complete the pharmacodynamic evaluation based on the target active component group is another key to the research of the spectrum–effect relationships of TCM. At present, animal, organ, cell, molecule, and other pharmacological models are often used in the pharmacodynamic study of specific disease models. Then, through appropriate stoichiometric methods, the fingerprints representing the compound information of TCM are combined with the pharmacodynamic information representing the pharmacodynamic index, which can complete the establishment of the spectrum–effect relationships. Therefore, this part mainly includes four points: samples with differences, establishment of fingerprints, evaluation based on pharmacodynamics and association based on chemometric methods.

### 2.1. Samples with Differences

**Figure 2:**
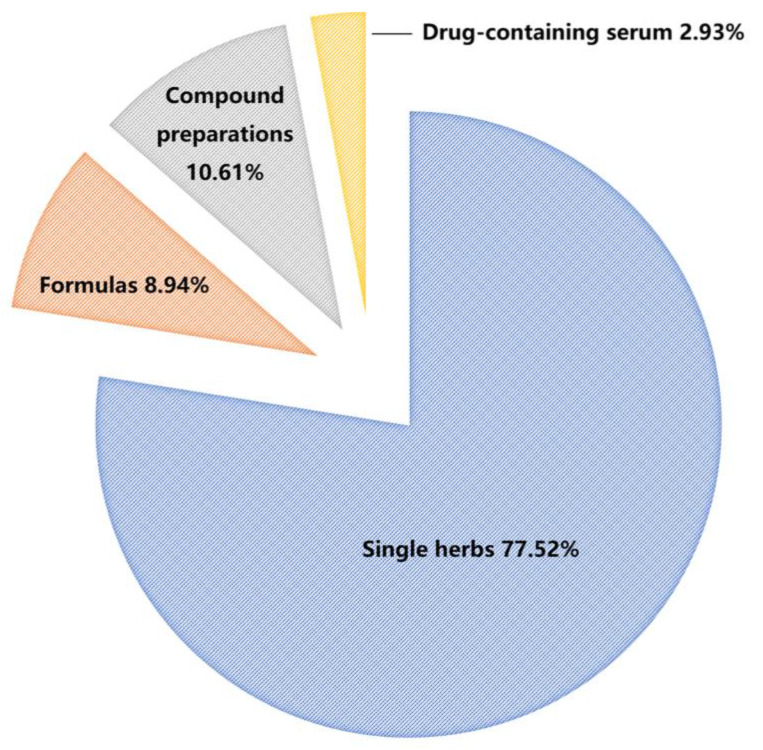
The proportion of the number of papers published by different research objects of spectrum–effect relationships.

Through the analysis of the published literature, it is found that single herbs have attracted much attention as the research hotspot, accounting for more than 70% of the research objects of the spectrum–effect relationships (Figure 2). At present, in the research on the spectrum–effect relationships of TCM, the samples are mostly from different batches, different cultivating regions or different manufacturers, and the samples from the same source can also be used to facilitate the follow-up difference treatment. For the samples with different sources, due to the differences in composition and efficacy, it is usually enough to use the same method to treat each sample separately. For example, different batches of *Artemisia frigida* Willd. [11] and Saposhnikoviae Radix [12] of different origins are pre-treated with the same method. In the process of sample pretreatment, it is the ultimate goal to select the effective parts and retain the effective components to the maximum extent [13]. Furthermore, for samples from a single source, sample pretreatment is mainly for purposeful manufacturing of component differences. For example, samples with differences are prepared by different extraction methods or processing methods, and the active component screening is completed by looking for differences in the spectral effect relationship between them [14,15].

For the spectrum–effect relationships of formulas and preparations of TCM, one or a group of drugs can be gradually removed from the formula, and then the efficacy of the remaining drugs can be investigated to obtain the efficacy of the removed drugs on the whole formula, through which the interaction of a single drug in the formula and preparation can be explored, as well as the best combination of multiple drugs can be found. For instance, Lan et al. revealed the qualitative and quantitative contributions of eight kinds of herbs in Zhizi Jinhua pills by prescription and decomposition analysis [16]. In addition, through the research of the spectrum–effect relationships of the formula and preparation from different batches, the main active components can also be discussed. Similar studies have been used in Banxia Baizhu Tianma decoction [17], compound licorice tablets [18] and Tianmeng oral liquid [19].

In addition, it is possible that the serum, after administration, is the “preparation” with the true curative effect. No matter in what form the drug is administered, the components that play a role are mainly those that enter the blood circulation of the body, and these components may be the material basis of the efficacy, which mainly include the prototype components of medicinal materials, metabolic substances and physiologically active substances. As early as 2002, research on the serum pharmacochemistry of TCM was reported by scholars [20]. For the study on the spectrum–effect relationships of the drug-containing serum, most of the samples with different component characteristics were obtained by blood collection at different times after administration, at different doses or at the same time after administration of different formulations [21,22]. Serum samples are usually pretreated by a single method. Since animal serum samples contain various endogenous proteins, which seriously interfere with the detection of components in blood, organic solvents, such as acetonitrile and methanol, are often directly added to precipitate proteins. Furthermore, in order to improve the detection sensitivity, extraction is often used to purify and enrich the samples. Moreover, sometimes, serum samples from different individuals are mixed to reduce individual differences.

### 2.2. Establishment of Fingerprints

After the completion of sample pretreatment, the selection of analysis methods for target components in TCM is particularly important. The analytical methods to establish TCM fingerprints mainly include (Ultra) high-performance liquid chromatography (HPLC or UHPLC), gas chromatography (GC), capillary electrophoresis (CE), thin layer chromatography (TLC), ultraviolet spectroscopy (UV), infrared spectroscopy (IR), and others. Among them, chromatography is the mainstream method, and HPLC has become recognized as the most conventional analytical method. Due to the characteristics of high separation efficiency, high selectivity, high detection sensitivity, fast analysis speed and wide application range, most TCM components can be analyzed and detected on HPLC, which has become the preferred method to establish TCM fingerprints. In the research of the spectrum–effect relationships, it is particularly important to select the appropriate analytical method because of the complexity and variety of TCM components.

Different types of compounds can be detected by connecting different types of detectors (UV/diode-array detector (DAD)/evaporative light-scattering detector (ELSD)) with HPLC [23,24], and multiple types of compounds can be detected by connecting different types of detectors in series. Therefore, it is most widely used in the detection of small and large molecular compounds. The GC-flame ionization detector (FID) has the advantages of high separation energy, high sensitivity, high selectivity, and less sample consumption [25,26]. It is suitable for the separation and analysis of volatile compounds that are not easy to decompose. Compared with HPLC, CE-UV can analyze not only small and medium molecular compounds but also large molecular compounds that are difficult to analyze by HPLC with faster speed and lower solvent consumption [27,28]. TLC is easy to obtain, simple, fast and cheap; however, TLC requires visual data acquisition, and it is not conducive to the construction of sample spectrum–effect relationships, so there are not many applications [29,30,31]. Compared with chromatographic analysis methods, UV [32] spectra mainly reflect the overall quality of compounds containing unsaturated chemical bonds and conjugated structures, and IR [33] spectra mainly reflect the functional group information of each component. Through spectral analysis methods, differences in the total component information of the whole substance between different samples can be found, but there is a shortcoming in that it is not possible to accurately characterize the different components. Based on this, the basic information of several commonly used analysis methods (including different detectors), including advantages and limitations, are summarized briefly in Table 1.

With the development of analytical instruments, high-resolution mass spectrometry (MS) with higher flux, stronger sensitivity and compound structure annotation function can be combined with the above techniques, especially HPLC-MS [34,35], GC-MS [36,37] and inductively coupled plasma (ICP)-MS [38,39], which could conduct qualitative and quantitative analysis of the intrinsic components of TCM more comprehensively and accurately. In addition, two-dimensional chromatography shows higher peak capacity and stronger separation and analysis ability, which makes it prominent in the analysis and screening of complex effective components of TCM [40].

In addition to developing analytical instruments, some researchers have proposed innovative concepts based on traditional techniques aimed at fully characterizing the overall chemical characteristics of complex material systems. Due to the complex composition of TCM, it is often difficult for a single chemical fingerprint established by one detector or one analytical method to accurately express its chemical composition characteristics. The concept of “multidimensional fingerprints“ of TCM has been proposed [41]. By using different sample pretreatment methods, different instrumental analysis methods or detectors to obtain multiple fingerprints can realize the complementary information and obtain more and complete sample information, and finally, obtain the fingerprints that can effectively represent the chemical characteristics of TCM. Moreover, in the use of HPLC-UV, the fingerprints established with a single detection wavelength may not be able to reflect the overall chemical composition information of the sample in a comprehensive and systematic way. In this case, the multi-wavelength fusion fingerprints method can make up for this limitation and achieve a more comprehensive analysis of the sample [42].

In general, different samples need to find the matching fingerprints as often as possible, and try to ensure more detailed characterization of chemical components to avoid missing important information of samples. After selecting an appropriate herbal sample analysis technique, proper pretreatment of the original data is the prerequisite for further data analysis, such as peak comparison, similarity analysis, data conversion, etc. [43,44]. As the first step to establishing the spectrum–effect relationships, comprehensive and detailed fingerprints could lay a solid foundation for the screening of the real active components.

### 2.3. Evaluation Based on Pharmacodynamics

**Figure 3:**
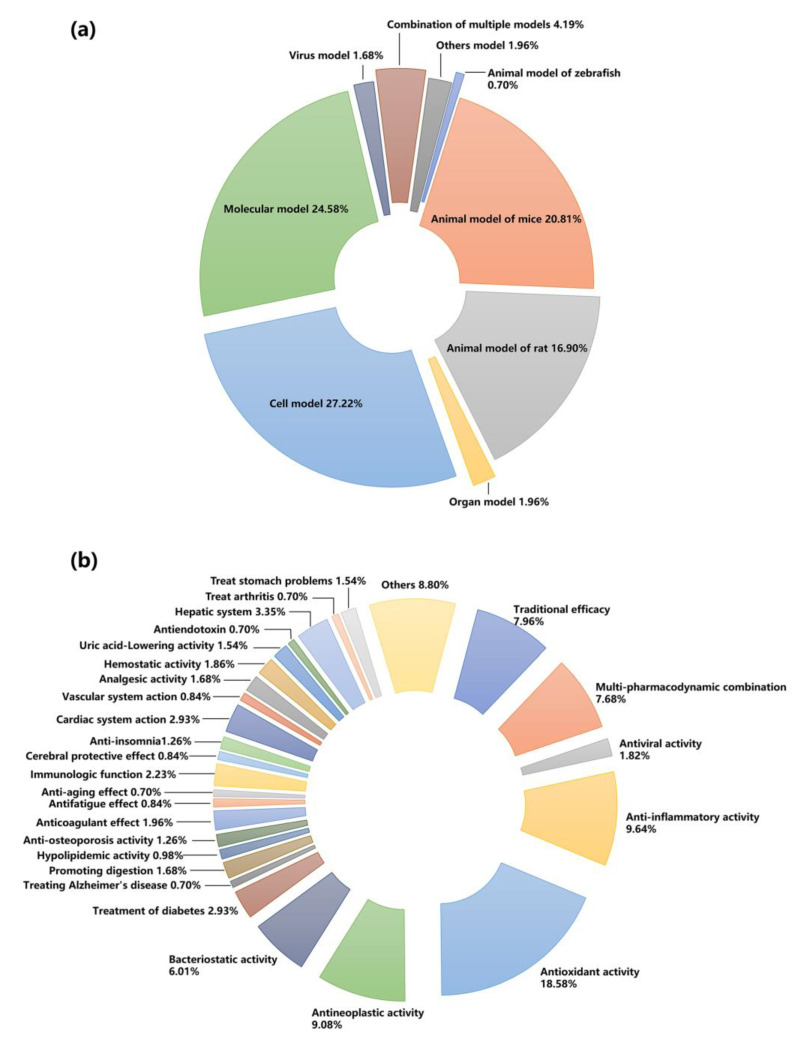
The proportion of the number of papers published by different types of pharmacological models (**a**) and different pharmacodynamic indexes (**b**) of spectrum–effect relationships.

The effects and mechanisms of TCM on the body are complex, and the synergistic effects of multi-components, multi-targets and multi-pathways determine that it is not a small challenge to find an appropriate pharmacodynamic evaluation method. How to establish an appropriate pharmacodynamic evaluation method can be summarized as follows: select an appropriate, rapid, and recognized pharmacodynamic evaluation model and pharmacodynamic indicators with high stability and good reproducibility for follow-up experiments. Animal models or in vitro organs are often used to observe the intuitive efficacy of drugs. Cell culture and biochemical experiments are usually used to study the mechanism of action. Herein, we summarize the application of different types of pharmacological models and different pharmacodynamic indexes in the study of spectrum–effect relationships in the past 20 years (Figure 3).

Current pharmacodynamic studies are mainly based on animal, organ, cell, molecular and other pharmacological models. Compared with the animal integrity level, the other three studies do not take into account the overall regulation of TCM on the body, nor the complex process of TCM absorption, metabolism, and distribution in the body. In addition, animals at the holistic level were more representative when responding to traditional efficacy such as “reinforce kidney to strengthening Yang” [24], “invigorating blood and dissolving stasis” [45], and “spleen-invigorating and anti-swelling” [46]. Therefore, at present, a large number of studies on spectrum–effect relationships have adopted the whole animal model, including rats, mice, zebrafish, etc. This model’s advantage is that it ensures the integrity of the body and the normal contact environment with the outside world. At the same time, this model aligns well with the subsequent clinical research. In the research of spectrum–effect relationships, as shown in Figure 3, the proportion of animal models was about 38.41%, higher than 1.96% for organ models, 27.22% for cell models, and 24.58% for molecular models respectively. However, the disadvantages of in vivo models are their high cost, long consumption time, and difficulty in establishing animal models with all the characteristics of human disease prototypes when the diseases under study have complex pathogenic mechanisms.

In vitro experiments at the organ, cell and molecular levels can directly reflect the pharmacodynamic activity of TCM because the target part can be separated from the complex in vivo environment. In addition, it can also alleviate the low-throughput problem of pharmacodynamic detection. These in vitro pharmacodynamic methods have been applied in many scenarios because of their low cost, short consumption time, good repeatability and easier to obtain perfect experimental data, such as nitric oxide (NO) inhibitory assays for measuring anti-inflammatory activity [37]; 3-(4,5-dimethylthiazol-2-thiazolyl)-2,5-diphenyltetrazolium bromide (MTT) assay for determining anti-bacterial activities [47]; and 1,1-diphenyl-2-trinitrophenylhydrazine (DPPH), 2,2’-diazide-bis (3-ethylbenzothiazoline-6-sulfonic acid) diammonium salt (ABTS), and ferric-reducing antioxidant power (FRAP) assay for evaluating antioxidant activity [48]. Therefore, this may also be one of the reasons why the application of antioxidant effects in the research of spectrum–effect relationships accounted for as high as 18.28% (Figure 3). Nevertheless, these single models also separate the overall role of the body, and there are still some limitations in many pathological models.

Due to the development of serum pharmacology, in addition to the above models, a series of experiments of drug-containing serum in vivo and in vitro are also a hot topic in the study of spectrum–effect relationships. The experimental animals were given TCM or formula one or more times, and blood samples were collected after a certain period of time [49]. The serum was used directly for chemical analysis or isolated to replace the crude extract of TCM or formula for in vitro pharmacological experiments. Based on this, the drug-containing serum could objectively simulate the process by which drugs produce pharmacological effects in the internal environment [8]. At the same time, based on a comprehensive analysis of drug-containing serum, pharmacodynamic experiments can be conducted to determine the real active components to explore the dynamic metabolism of active components. However, there are many problems in the research of drug-containing serum, as the sample pretreatment process is relatively complicated, which can easily cause the loss of components and lead to the deviation of experimental results. Furthermore, it consumes a large amount of blood and is unsuitable for small animals, and it is difficult to evaluate the impact on animal models, which may introduce a significant error [49].

Pharmacodynamic indicators are an important standard to measure the efficacy of TCM. Currently, one or several pharmacodynamic indicators are used to evaluate the spectrum–effect relationships. As can be seen from Figure 3b, the commonly used pharmacodynamic indexes in the research of spectrum–effect relationship include antioxidant (18.58%), anti-inflammatory (9.64%), antineoplastic (9.08%), and antineoplastic activities (6.01%). Furthermore, to ensure the comprehensiveness of pharmacodynamics, some scholars have proposed the inclusion of multi-index pharmacodynamics into the study of spectrum–effect relationships [41,50]. It should be noted that although multiple pharmacodynamic indicators can complement and corroborate each other, they sometimes lead to the opposite. In view of the different processes of TCM in the treatment of diseases, the manifestations of different components in the symptoms and root causes of diseases may be different, so clarifying the pathogenesis of diseases and clarifying the mechanism and target of drugs is conducive to the selection of correct and appropriate pharmacodynamic indicators. In general, the key is to select targeted indicators that can represent the main efficacy of TCM.

### 2.4. Association Based on Chemometric Methods

It is another important link in the study of spectrum–effect relationships to realize the correlation between the obtained fingerprints and pharmacodynamic information of TCM and to dig out the information we want from them. At present, a variety of chemometric methods have been applied to the research of spectrum–effect relationships, including grey relational analysis (GRA), bivariate correlation analysis (BCA), hierarchical clustering analysis (HCA), artificial neural networks (ANNs), multivariate linear regression (MLR) analysis, partial least-squares regression (PLSR) analysis, canonical correlation analysis (CCA), principal components analysis (PCA) and other methods.

Different chemometric methods have their own emphasis directions. By selecting the appropriate data analysis method, the accurate analysis of the pharmacodynamic mechanism in the complex TCM matrix can be completed more effectively. At the same time, to ensure the accuracy of analysis results, many researchers combine two or several chemometric methods to select methods for the study of spectrum–effect relationships to achieve the effect of comprehensive analysis. The characteristics, advantages, and disadvantages of each data analysis method are summarized in Table 2. In addition, these methods can also be summarized into three categories: methods to predict the correlation between each component and efficacy, methods to clarify the contribution of each component to efficacy, and methods to find the main active component by simplifying data structures [51].

#### 2.4.1. Methods to Predict the Correlation between Each Component and Efficacy

The correlation between pharmacodynamic indexes and chromatographic peaks can be calculated by GRA, BCA, HCA and ANNs methods, which provides a possibility for predicting active components. GRA is based on the degree of similarity between the geometric shapes of curves between variables to determine the correlation between variables, which is especially suitable for complex variables involving less information [52]. In addition, the information available to GRA can be used to predict the information not yet available. Still, it is difficult to describe the overall contribution of the corresponding components of each peak.

BCA is one of the most widely used correlation chemometric methods in studying spectrum–effect relationships. By calculating the correlation coefficient between two variables, it studies the nature and closeness of the relationship between one variable and another variable [53]. The most commonly used correlation coefficient is Pearson’s, which reflects the degree of correlation between two variables and reflects the positive or negative direction of them. Unfortunately, it also ignores the integrity of TCM and cannot explain the synergistic effect of various peaks on pharmacodynamics.

Based on the principle that “like attracts like”, HCA, as the most widely used data analysis method in cluster analysis [54], is often used to analyze the fingerprints of different samples, then select appropriate sample groups based on the results, and combine with other chemometric methods to study the spectrum–effect relationships. However, HCA cannot reflect the magnitude and direction of the correlation between fingerprint peaks and pharmacodynamic indexes. Therefore, it is usually used as the preliminary analysis before the actual modeling.

ANNs is a mathematical modeling algorithm that simulates the signal transmission mode of an animal neural network and carries out information processing. In addition, the backpropagation (BP) ANN is one of the most widely used multi-layer feedforward networks based on the BP algorithm in spectrum–effect relationships research. It can use the chemical component signals in the existing fingerprints, extract complex information effectively through conversion and compression and establish the fuzzy mapping function relationship between these signals and the corresponding pharmacodynamic indexes. However, its principle is based on empirical risk minimization, which may lead to the risk of overfitting for small amounts of sample data [55].

#### 2.4.2. Methods to Clarify the Contribution of Each Component to Efficacy

It is difficult for the above four chemometric methods to describe the contribution rate of each component in the fingerprints to efficacy. The regression model of each peak and pharmacodynamic data can be established by the regression analysis method to measure the contribution rate of active components to the pharmacodynamic data, among which MLR and PLSR are the two commonly used methods.

By establishing regression models with multiple independent variables and a single variable, MLR performs a parameter evaluation on the impact of each independent variable on the dependent variable, which is a useful method to quantify the relationship between spectrum and bioactivities [33]. When establishing the linear relationship between the dependent variable and independent variable, stepwise regression is often adopted to complete the screening of the independent variable to simplify the computational complexity; however, the pharmacodynamic relationship of chromatographic peaks not selected into the model will be ignored. However, when there are multiple linear relationships between the independent variables or the number of samples is small, MLR cannot guarantee the accuracy and reliability of the established spectral effect model, in which case PLSR can be used [56].

When the internal variables are highly linearly correlated, PLSR is more effective and can better solve the problem that the number of samples is less than the number of variables. It not only makes maximum use of data information but also has the advantages of small computation, high prediction accuracy, and easy qualitative interpretation [57]. Moreover, in orthogonal projections to latent structure (OPLS), the first latent variable is calculated using a linear combination of manifest variables defined in the direction of the highest co-variance with the response. The following latent variables are defined in the direction of the remaining variance (not co-variance) around the first, the plain between the first and second. The discriminant analysis (OPLS-DA) model between the chemical composition expression and the sample class is used to realize the prediction of the sample class, which is suitable for the separation between the two groups of samples and the mining of differential variables. The contribution of variables to the model can be explored through the variable importance in projection (VIP) value. The larger the VIP value (generally more than 1.0), the greater the contribution [58,59]. By screening the VIP values of different compounds, it is easy to find active compounds with high correlation coefficients with pharmacological indexes.

#### 2.4.3. Methods to Find the Main Active Component by Simplifying Data Structure

Due to the complexity of the chemical composition of TCM, many complicated variables are interrelated, so it is not easy to find the important effective ingredient. Both CCA and PCA use the idea of dimensionality reduction to combine the original multiple index information into a few comprehensive indicators for analysis so as to judge the correlation degree of the original variables according to the comprehensive indicators. In short, they could reduce the original multiple-dimension data into two-dimensional or three-dimensional data for analysis to achieve the purpose of data simplification [51]. The two most representative comprehensive variables (the linear combination of variables in the two variable groups) are extracted from the two groups of variables, the canonical variables. CCA can reflect the overall correlation between the two groups of indicators by using the correlation between the two canonical variables. However, although it simplifies complex data, the data information after dimensionality reduction is also reduced [60].

PCA uses dimension reduction technology to replace the original multiple variables with a few comprehensive variables, namely principal components (PCs), which preserve most of the information of the original variables. In general, the number of PCs is determined by the cumulative contribution rate and the size of the eigenvalue; the cumulative contribution rate of >85% and the eigenvalue of λi ≥ 1 is appropriate. However, PCA cannot quantitatively analyze the correlation between variables through a mathematical model, so it is often combined with other chemometric methods such as cluster analysis, correlation analysis or regression analysis in the research of spectrum–effect relationships [22]. Both PCA and CCA extract different information by constructing appropriate linear combinations of the original variables; PCA only involves the interdependence of a group of variables, while CCA extends to the interdependence of two groups of variables and measures the strength of the relationship between the two groups of variables.

According to the classification of the above chemometric methods, it can be found that various chemometric methods have their own characteristics. The common thread in these methods is an effort to find ingredients that correlate with efficacy. Given the advantages and disadvantages of various data analysis techniques and their characteristics, these methods should be comprehensively applied and cross-verified to achieve the purpose of promoting the research of the spectrum–effect relationships in TCM.

## 3. Applications of Spectrum–Effect Relationships

### 3.1. Quality Evaluation of TCM

The quality of natural products such as TCM can be evaluated scientifically through the accurate analysis of the pharmacodynamic components contained in TCM and its compound preparations by using the spectrum–effect relationships (Table 3). Most TCM comes from natural products grown or cultivated in the wild and can be divided into different specification grades according to traditional empirical criteria, such as their shape, color, odor, and taste. The commodity specification grade of Chinese medicinal materials is not only a representative sign of the quality of TCM but also one of the bases of its price in the trading and circulation link [61]. An et al. used this method to analyze three grades of Sennae Folium with different specifications. They found that the first premium grade was a green leaf, followed by a yellow leaf and diseased leaf. They identified that six sennosides might be the material bases for in vitro antibacterial activity and a marker for grade differentiation [58]. Similarly, this pattern has also been successfully applied to studying the spectrum–effect relationships of Notoginseng [62] of twelve different commodity specifications and Notopterygii Rhizoma [63] of three commercial specifications. Through the study of the spectrum–effect relationships between different specifications of TCM, it is helpful to provide a reference for the classification of TCM commodity specifications.

Under the joint action of plant genotype, specific ecological environment, and cultivation measures, authentic medicinal materials were formed according to their origin [64]. Multi-origin planting is one of the characteristics of Chinese medicinal materials, and the study of spectrum–effect relationships can be used for quality control of TCM from different origins. Ten batches of Saposhnikoviae Radix from four different origins in China were used to research the spectrum–effect relationships between 1-phenyl-3-methyl-5-pyrazolone (PMP)-HPLC, Fourier transform infrared spectroscopy (FT-IR) and high-performance size-exclusion chromatography (HPSEC) fingerprints and the anti-allergic activity of their polysaccharides. It was confirmed that the materials harvested from Inner Mongolia had the best anti-allergic activity [12]. In addition, Jiang et al. developed a method for assessing the quality of Chinese propolis from different geographical sources by studying the correlation between the chemical composition and its anti-DPPH activities in 49 samples from different regions of China [56]. Furthermore, TCM has a variety of different species, and the same plant has a variety of medicinal parts. Although the chemical fingerprint characteristics of these different varieties and different medicinal parts are generally similar, their pharmacodynamic basis can be further determined by spectrum–effect relationships. In recent years, some scholars have studied the spectrum–effect relationships of different species of Rhizoma Coptidis [65], Chrysanthemum morifolium [66] and Zicao [67], respectively, and also explored the material basis and activity of different drug parts of *Morus alba* L. [68] and Trichosanthis Semen [69].

In addition to the quality evaluation of a single herb, the spectrum–effect relationship can also be used to determine the main active substances in formulas and preparations of TCM to evaluate their quality by focusing on the main active substances. The difficulty in researching the spectrum–effect relationships between formulas and preparations lies in the high throughput of compound fingerprints and the selection of a pharmacodynamic model based on traditional efficacy. With the development of analytical instruments and pharmacodynamic models, more reports have been on the spectrum–effect relationships between formulas and preparations in the past five years. Chinese medicine herbal pair refers to the common application of two kinds of TCM, the relatively fixed minimum prescription unit in the formulas. Among them, the Danshen-Honghua herbal pair, which is composed of Salvia miltiorrhiza and Carthamus tinctorius, is a combination of TCM with the effect of blood-activating, and their combination has been widely used in the treatment of cardiovascular diseases [70]. In recent years, some scholars have systematically studied anti-vascular dementia [71], tyrosinase inhibition [72] and blood-activating effects [73] of Danshen-Honghua herbal pair by spectrum–effect relationships.

In some formulas covering more kinds of TCM, the spectrum–effect relationships have also been successfully applied to the discovery of pharmacodynamic components of various compounds, such as Banxia Baizhu Tianma Decoction [17], Banxia Xiexin Decoction [74], Si Jun Zi Tang [75], etc. The Qi-Yu-San-Long decoction (QYSLD) is a TCM formula composed of ten kinds of herbs, which is mainly used in the clinical treatment of non-small cell lung cancer (NSCLC) [76]. Huang et al. used three chemometric methods, including GRA, PLSR and BPANN, to establish the UHPLC-Q/TOF-MS fingerprints of QYSLD and correlated it with the antioxidant and anti-NSCLC effects of the formula and found different material bases associated with different activities. Finally, compared with the unscreened ingredients, the potential active ingredients obtained by spectrum–effect relationships have better efficacy through the confirmatory experiment [55]. In addition to formulas, the research of spectrum–effect relationships can also be used to guide the establishment of a quality evaluation system for modern Chinese medicine preparation. Lan et al. established an intelligent model through spectrum–effect relationships to analyze the contribution of eight single herbs in Zhizi Jinhua pills [16]. By combining a variety of active ingredients of antioxidant activity, the quality monitoring method based on fingerprint contour control is realized. Moreover, a research used Yindan Xinnaotong soft capsule to inhibit microglia-mediated neuroinflammation to establish an image-based fingerprints-efficacy screening strategy [77]. It was found that tanshinones and flavonoids were potential anti-neuroinflammatory compounds in the preparation. Furthermore, many achievements have been made in the quality evaluation of Xinkeshu Tablets [78], Rong’e Yishen oral liquid [79] and other TCM preparations by spectrum–effect relationships.

**Table 3:** Application of spectrum–effect relationships in quality evaluation of TCM.

Objective	TCM	Fingerprints	Pharmacodynamic	Chemometrics	Component	Reference
Grade	Sennae Folium	UHPLC-Q-TOF/MS	Laxative effect (lipase inhibitory activity)	PCA, OPLS-DA	8 sennosides and anthraquinones, 11 flavonoids, and 3 benzophenones	[58]
	Notoginseng	HPLC	Hemostatic effects (rat plasma recalcification experiment and the rat gastric bleeding experiment)	PLSR	Notoginsenoside R_1_, Ft_1_, and ginsenoside Rg_1_, Re, Rg_2_, Rh_1_, F1, Rk_1_, and dencichine, and unconfirmed peak 4, 6, 8, 9, 13, 14, 15	[62]
	Notopterygii Rhizoma	HPLC	Anti-inflammatory activity (xylene-induced acute inflammation model of mouse ear swelling and lipopolysaccharide (LPS) induced mononuclear macrophage RAW264.7 model)	GRA	17 components, including chlorogenic acid, ferulic acid and isoimperatorin	[63]
Origin	Saposhnikoviae Radix	PMP-HPLC, FT-IR, and HPSEC	Anti-allergic activity (MTT assay of RBL-2H3 cells)	GRA	Two monosaccharides (rhamnose and galactose), the polysaccharide fragment Mn = 8.67 × 10^6^~9.56 × 10^6^ Da, and the FT-IR absorption peak of 892 cm^−1^	[12]
	Chinese propolis	HPLC	Antioxidant activity (DPPH assay)	PCA, MLR	Isoferulic acid, caffeic acid, caffeic acid phenethyl ester, 3,4-dimethoxycinnamic acid, chrysin and apigenin	[56]
Species	Rhizoma Coptidis	UHPLC/QqQ-MS	Treating Alzheimer’s disease (anti-acetylcholinesterase activity)	Random forest, Boruta and Pearson correlation	Columbamine, berberine and palmatine	[65]
	Chrysanthemum morifolium	HPLC	Antioxidant activity (DPPH, OH and ABTS assay)	PCA	Chlorogenic acid, 3,5-*O*-dicaffeoylquinic acid, unknown peak 1, 4,5-*O*-dicaffeoylquinic acid and kaempferol-3-*O*-rutinoside	[66]
	Zicao	UHPLC-QTRAP-MS/MS	Anti-tumor activity (HeLa cells)	OPLS	27 components, including shikonins, shikonofurans and β, β-dimethylacrylshikonin	[67]
Medicinal parts	*Morus alba* L.	HPLC	Antidiabetic activity (α-glucosidase inhibitory activity)	OPLS	Morin, sanggenon C, kuwanon G, morusin, kaempferol, quercetin, rutin, isoquercitrin, and 1-deoxynojirimycin	[68]
	Trichosanthis Semen	HPLC	Anticoagulant activity (prothrombin time and activated partial thromboplastin time in mice)	Deng’s correlation degree	Adenine, uracil, hypoxanthine, xanthine and adenosine	[69]
Formulas	Danshen-Honghua herbal pair	HPLC	Therapeutic effect on vascular dementia (alleviated phenylhydrazine-induced thrombosis and improved bisphenol F and ponatinib induced brain injury in zebrafish)	PLSR	Danshensu, hydroxysafflor yellow A, kaempferol-3-*O*-rutinoside, rosmarinic acid, lithospermic acid, salvianolic acid B, salvianolic acid A, dihydrotanshinone I, cryptotanshinone, tanshinone I, and tanshinone IIA	[71]
	Banxia Baizhu Tianma Decoction	UHPLC	Antioxidant and anti-inflammatory activities (reactive oxygen species and high sensitivity C-reactive protein models)	HCA and BCA	Gastrodin, liquiritin, hesperidin, isoliquiritin, hesperidin, and isoliquiritigenin	[17]
	Si Jun Zi Tang	UPLC	Antiproliferative effect (PC9 cells)	HCA and CCA	Ginsenoside Ro and ginsenoside Rg_1_	[75]
	Qi-Yu-San-Long decoction	UHPLC-Q/TOF-MS	Antioxidant activity (DPPH and FRAP assay) inhibits the proliferation ability, horizontal migration ability, vertical migration ability and invasion ability of A549 cells	GRA, PLSR and BPANN	Eight, nine, six, twenty-two, five, and twelve ingredients correspond to 6 therapeutic effects, respectively	[55]
Preparations	Zhizi Jinhua pills	HPLC	Antioxidant activity (SOD and MDA in mice serum; DPPH and ABTS assay)	OPLS	24 of the 30 fingerprints peaks	[16]
	Yindan Xinnaotong soft capsule	LC-MS and GC-MS	Anti-neuroinflammatory activity (inhibit microglia-mediated neuroinflammation)	Pearson correlation analysis	Scutellarin, apigenin-7-*O*-glucuronide, scutellarein, apigenin, przewaquinone A, dihydrotanshinone I, tanshinone I, cryptotanshinone, tanshinone IIA and miltirone	[77]
	Xinkeshu Tablets	HPLC	Antiarrhythmic effect (heart rate recovery rate of zebrafish larvae)	OPLS	Danshensu, salvianolic acid A, salvianolic acid B, daidzein, and puerarin	[78]
	Rong’e Yishen oral liquid	HPLC and electrochemical fingerprints	Antioxidant activity (ABTs)	PLSR and BCA	15 components of the 48 co-possessing peaks	[79]

### 3.2. Pharmaceutical Process Optimization of TCM

The spectrum–effect relationships could not only be used for the quality evaluation of TCM and compound preparations but also provide an evaluation system for the optimization of pharmaceutical process optimization of TCM. Through spectrum–effect relationships research, the discovery and tracking of the active ingredients could be realized to guide the optimization process of pharmaceutical technology, which could make maximum protection of the active ingredients and reduce the potentially toxic substances so as to achieve the purpose of maximizing the clinical efficacy of TCM. In practical applications, the effective fractions and the extraction process can be screened and optimized according to the properties of the active ingredients (Table 4). Aurantii Fructus is a commonly used qi-regulating medicinal herb in China, widely used to treat gastrointestinal motility disorders (GMD). Qiao et al. systematically compared the effects of five fractions of Aurantii Fructus, including petroleum ether, chloroform, ethyl acetate, n-butanol and water, on normal mice and GMD rats. They found the ethyl acetate fractions with the strongest regulating-qi effect through the spectrum–effect relationships, among which nine compounds were the main regulating-qi components [80]. However, researchers have found strong dryness in Aurantii Fructus, which may be harmful to the body fluids [81]. In order to further explore the dryness of different fractions of Aurantii Fructus and the material basis of dryness, based on the above research, a spectrum–effect relationship revealed that ethyl acetate and petroleum ether fractions were the main sources of dryness effect caused by Aurantii Fructus in model rats [82]. In addition, some scholars have studied the spectral effect relationship of various TCMs, such as Callicarpa nudiflora [83], Radix Hedysari [84], and propolis [85], and determined the location of the active components of the main fractions.

Combined with a single-factor experiment, the scavenging ability of *Angelica dahurica* on free radicals under different extraction conditions was compared to obtain the optimal extraction conditions [86]. The results provided a reference for the effective utilization of antioxidant components of *Angelica dahurica*. With the emergence of novel extraction technologies, there may be room for improvement in the extraction efficiency of effective substances compared with traditional extraction methods. A microwave-assisted extraction method for the anti-diabetic active ingredients of *Nigella glandulifera* Freyn was developed based on the spectrum–effect relationships [87]. Compared with the traditional extraction method, this method has a shorter time, higher yield, and higher biological activity.

Processing (Paozhi) represents a unique Chinese pharmaceutic technique to facilitate the use of Chinese herbal medicines for a specific clinical need in the guidance of TCM theory [88]. After the processing of TCM, it can enhance the efficacy and reduce the toxicity, such as the anti-osteoporosis efficacy of Radix Dipsaci [89], which could be strengthened after salt-processed, and the toxicity can be reduced after the processing of *Polygonum multiflorum* Thunb. [90]. Through the research of the spectrum–effect relationships of the samples before and after processing, the substances related to the changes of curative effect can be found accurately so as to explain the change of efficacy of TCM before and after processing. Yang et al. established, for the first time, a spectrum–effect relationship between the antitussive, expectorant and anti-inflammatory activity of raw and honey-processed Farfarae Flos samples based on UPLC and proved that both raw and processed Farfarae Flos had antitussive and anti-inflammatory effects, and only after processing did it have expectorant effects [91]. In a report, the chemical properties and neuroprotective/antioxidant activities of Panax Notoginseng under different steaming parameters were combined to establish a method based on spectrum–effect relationships to analyze the changes of TCM components during processing [92]. In addition, the spectrum–effect relationships between raw Panax Notoginseng and steamed Panax Notoginseng have also been systematically studied in terms of their anti-inflammatory, anticoagulant, and antioxidant activities [93,94].

**Table 4:** Application of spectrum–effect relationships in exploration of pharmaceutical process optimization of TCM.

Objective	TCM	Fingerprints	Pharmacodynamic	Chemometrics	Optimization Result	Reference
Extract fractions	Aurantii Fructus	UHPLC-Q-TOF/MS	Promoting gastrointestinal motility activity (normal mice and Gastrointestinal motility disorders model rats)	PCA, BCA and OPLS	Ethyl acetate fraction is the main active fraction	[80]
	Callicarpa nudiflora	HPLC	Anti-inflammatory effect (toes swelling in inflammatory rats)	Pearson analysis and OPLS	Extracts 1, 2, 3, and 5 had greater inhibition effects than the control group	[83]
	Radix Hedysari	HPLC	Treatment for osteoporosis (increasing the peak bone mass of rat)	GRA	The total extract of Radix Hedysari is the most effective extract	[84]
	Propolis	UHPLC-MS	Antioxidant activity (DPPH and ABTS assay); antimicrobial activity (Gram-positive, Gram-negative bacteria, and fungi)	PLSR	50% ethanolic extracts ensure a good antioxidant capacity; all extracts demonstrated antibacterial and antifungal activity	[85]
Extract conditions	*Angelica dahurica*	GC–MS	Antioxidant activity (DPPH, FRAP and ABTS assay);	HCA, PCA and PLSR	Ultrasonic extraction, 80% methanol, 20:1 (ml/g) ratio of liquid to material, and extraction time of 30 min	[86]
	*Nigella glandulifera* Freyn	HPLC	Anti-diabetic efficacy (In vitro antioxidant activity, Aldose reductase assay, PTP1B assays)	GRA	Microwave-assisted extraction, solid-to-liquid ratio of 1:20 g/mL, ethanol concentration of 70%, extraction time of 35.00 min, and extraction temperature of 66 °C	[87]
Processing conditions	Farfarae Flos	UPLC	Antitussive (cough in mice caused by ammonia liquor); expectorant (intraperitoneal injection of phenol red by mice); anti-inflammatory effects (degree of swelling of the mouse ear after application of xylene mice)	GRA and PLSR	Only after honey processing can Farfarae Flos have an expectorant effect	[91]
	Panax notoginseng	HPLC	Anti-Alzheimer’s disease activity (neuroprotective activity of samples by reducing the cytotoxicity of Aβ_1-42_ in PC12 cells); antioxidant effect (oxygen radical absorption capacity)	PLSR	Better anti-Alzheimer’s disease activity on neuroprotective effect and antioxidant effect can be obtained at a shorter steaming time and a higher steaming temperature	[92]
	*Polygonum multiflorum*	UPLC-Q-TOF-MS	Hepatotoxicity (L02 and HepG2 hepatocytes)	GRA, OPLS and BPANN	Emodin dianthrones, emodin-8-*O*-β-D-glucopyranoside, and PM 14-17 could be used as toxicity markers	[90]
	*Morinda officinalis*	HPLC	Protective effects against reproductive oxidative stress damage (cyclophosphamide-induced male mice)	GRA	F-fructofuranosylnystose, nystose, 1-kestoses, inulin-oligosaccharides and inulo-oligosaccharides could be considered Q-markers for processed products	[14]
Formulas and preparations process conditions	Qin Jin Hua Tan Tang	UHPLC	Anti-inflammatory activity (xylene-induced ear-swelling mouse model)	GRA, PLSR and redundancy analysis	Ethanol extract 1 exhibited good anti-inflammatory activity	[95]
	Radix Polygoni multiflori-Achyranthes bidentate	HPLC	Antiosteoporosis Effect (mice induced by retinoic acid)	MLR	The order of activity of extracts from each group was petroleum ether > water > ethanol > ethyl acetate > methanol > acetone	[96]
	Niuhuang Shangqing Pill	2D-LC	Antibacterial constituents (Anti-streptococcus pneumoniae)	PCA, OPLS and OPLS-DA	All the fractions had different degrees of anti-streptococcus pneumoniae effect, and the 60% ethanol fraction had the strongest effect	[40]
	Yuanhu Zhitong prescription	UPLC	Anti-alcoholic gastric ulcer (gastric lesion mice induced by anhydrous ethanol)	GRA and PLSR	Yuanhu Zhitong prescription made with vinegar treatment Corydalis Rhizoma had the strongest effect	[97]
Component compatibility	Lichong Shengsui Yin	HPLC	Anti-ovarian cancer activity (in vitro tumor inhibition experiments and the survival extension rate in tumor-bearing nude mice)	Regression analysis (ENTER Method, STEPWISE Method) and correlation analysis	The combination of group 3 and group 9 had anti-ovarian cancer activity	[98]
	Salviae Miltiorrhizae Radix et Rhizoma-Chuanxiong Rhizoma	HPLC	Thrombin inhibitory activity (THR and factor Xa inhibitory activity assay)	CCA	The 1:1 ratios of Salviae Miltiorrhizae Radix et Rhizoma–Chuanxiong Rhizoma herbal pairs show the strongest inhibition factor Xa	[99]
	Curcumae Rhizoma-Sparganii Rhizoma	HPLC	Anti-tumor activity (A549, HepG2, Hela, BGC-823, and MCF-7 cells)	Random forest	The 80% ethanol elution fraction showed strong anti-tumor effects	[100]
	Zhusha Anshen Pill	HPLC	Sedative-hypnotic effect (spontaneous locomotor activity test and pentobarbital-induced sleeping test of mice)	MLR and GRA	Fe^2+^ instead of Hg^2+^ to improve Zhusha Anshen Pill to create Tieshuang Anshen Prescription	[101]

There are also reports on the spectrum–effect relationships of the reduction of hepatotoxicity of *Polygonum multiflorum* after processing. Based on the theory of toxicity attenuation after processing, combined with the UPLC-Q-TOF-MS method and plant metabolomics, the different components reduced between raw and processed *Polygonum multiflorum* were screened. The spectrum–effect relationships analysis of the hepatotoxicity of the samples was conducted by LO2 and HepG2 hepatocytes, and it was found that sixteen components may have different degrees of hepatotoxicity; among them, emodin dianthrones, emodin-8-*O*-β-D-glucopyranoside, PM 14-17 could be used as toxicity markers, which could provide reference for the processing technology of *Polygonum multiflorum* to reduce toxicity [90]. The different processing products of *Morinda officinalis* (MO) have different clinical applications, although they come from the same medicinal herb. Liu et al. established chemical fingerprints of raw MO, steaming MO and salt-steaming MO by HPLC, evaluated the active ingredients in different processed products to protect against reproductive oxidative stress damage, and found that five oligosaccharides could be used as quality markers during processing [14]. Through the spectrum–effect relationships, these studies deepen the scholars’ understanding of the processing principle in the processing process and provide a scientific guide for the processing technology.

In addition to a single herb, the spectrum–effect relationships can also be used to optimize the process of TCM compound preparation. In recent years, the spectrum–effect relationships have been successfully applied to the optimization of the extraction process of various formulae, such as the anti-inflammatory activity of different polar fractions of Qin Jin Hua Tan Tang [95] and the anti-osteoporosis effect of different extract solvents of Radix Polygoni multiflori-Achyranthes bidentate [96]. In the process optimization of preparations, Wu et al. used two-dimensional liquid chromatography (2D-LC) to compare the anti-streptococcus pneumoniae activities of different fractions of Niuhuang Shangqing Pill, including 5%, 30%, 60% and 95% ethanol fractions. They determined that 60% ethanol fraction had the strongest activity [40]. Among them, compound X17, wogonin, glycyrrhizic acid and baicalein are the main antibacterial active components of the preparation. Moreover, the different components can be discovered through the spectrum–effect relationships when the herbs are processed and then added to the formulas. Wang et al. studied the anti-alcoholic gastric ulcer effect of Yuanhu Zhitong prescription (Corydalis Rhizoma and Angelicae Dahuricae Radix) before and after vinegar treatment based on the spectrum–effect relationships [97]. Compared with the model group, the lesions of mice in each administration group were relieved and treated to different degrees, and the Yuanhu Zhitong prescription made with vinegar treatment Corydalis Rhizoma had the strongest effect.

The study of component compatibility is a novel model in the study of formulas. The application of spectrum–effect relationships in the study of component compatibility could optimize the compatibility by studying the active substances in formulas. Wang et al. prepared four kinds of Lichong Shengsui Yin extracts by different extraction methods and then mixed each extract in different proportions to prepare nine kinds of mixture and compared the anti-ovarian cancer activity of different component compatibility and the main active ingredients to play the efficacy through the spectrum–effect relationships [98]. Similar studies have also been conducted on the optimization of nine different ratios of Salviae Miltiorrhizae Radix et Rhizoma–Chuanxiong Rhizoma herbal pairs (1:0, 1:1, 2:1, 3:1, 5:1, 1:5, 1:3, 1:2 and 0:1) [99], and the optimization of extracts of Curcumae Rhizoma–Sparganii Rhizoma with different polar elution fractions (water, 20%, 40%, 60%, 80%, and 95% ethanol) [100]. In the optimization of component compatibility, one study used Fe^2+^ instead of Hg^2+^ to improve Zhusha Anshen Pill to create Tieshuang Anshen Prescription, which not only improved the sedative and hypnotic effect of the compound but also avoided the toxicity of Hg^2+^ in the previous prescription [101].

### 3.3. Exploration of Drug-Containing Serum of TCM

The direct analysis method of serum fingerprints can exclude the influence of body metabolism on TCM, which could better reflect the pharmacodynamic material basis of preparations and is a more effective means to determine the pharmacodynamic material basis (Table 5). Black ginseng is a processed product of ginseng. Rats were administered intragastrically with black ginseng extract to obtain the drug-containing serum. The anti-proliferation effect of prostate cancer cells has been detected by MMT assay, and the effective components of anti-prostate cancer in black ginseng have been confirmed by spectrum–effect relationships [102]. Zhang et al. determined the plasma fingerprints of rats with middle cerebral artery occlusion dosed with Yangyin Tongnao Granules at different time points by HPLC. They detected the changes in the levels of inflammatory factors under medicated plasma at different time points [103]. Six components correlate with the inflammation index by studying spectrum- effect relationships. The strategy based on the spectrum–effect relationships of the drug-containing serum is also used to screen the effective components of the protective effect on the glomerulus of Fangji Huangqi Tang [21] and the anti-hepatoma effect of Dahuang Zhechong Pill [104]. In addition, the spectrum–effect relationships can also be applied to the research of toxic or side-effect-related components of TCM to control the quality it from the perspective of drug safety. Shuang Huang Lian injection is an effective Chinese medicine in the treatment of acute respiratory tract infection, but it causes many severe anaphylactic reactions. By injecting the preparation into the caudal vein of rats, serum samples were obtained in vivo, and the spectrum–effect relationship was studied [105]. Chlorogenic and other components were identified as anaphylactoid components in the preparation, which guided the screening of allergic components in other TCM preparations.

## 4. Novel Strategy in Spectrum–Effect Relationships

With the continuous development of the study of the spectrum–effect relationships, its research system has been further extended, and a series of new strategies for TCM quality evaluation models have been developed. Some studies are not satisfied with the active compounds obtained by using the spectrum–effect relationships criterion but will further verify the pharmacodynamic activity of the compounds in the early results to ensure the accuracy of the final results. Furthermore, the combination of spectrum–effect relationships with network pharmacology and molecular docking techniques can elucidate the mechanism of action of the obtained active compounds, which points out the direction for the subsequent study of pharmacodynamic mechanism. Moreover, QAMS techniques can also be combined with current research strategies to contribute to the quantitative analysis of active compounds.

### 4.1. Efficacy Verification by Monomeric Compound and Component Knock-Out

In the study of spectrum–effect relationships, some chromatographic peaks with pharmacodynamic activity are screened by stoichiometric methods, and researchers often complete the qualitative analysis of compounds by the retention time of standard substances, mass spectrometry identification and other technologies to verify the accuracy of the results. However, there are still some cases where the chromatographic peaks fail to achieve structural identification, which is defective for the results of spectrum–effect relationships. For active ingredients whose structures have been determined, efficacy tests can be conducted again by recombining single or multiple selected ingredients to verify whether there is bioactive equivalence between the candidate active ingredients and the original TCM, which can be referred to existing reports [106,107]. In recent years, another efficacy verification strategy based on the “knock-out” method has been applied to study spectrum–effect relationships. After determining the pharmacodynamic substances of TCM, the active substances are removed from the TCM by component “knock-out”, and the pharmacodynamic changes of TCM after the removal of the pharmacodynamic substances are investigated [108,109]. In this way, the pharmacodynamic role of the compounds have pharmacodynamic characteristics in TCM is further clarified, which is also a supplement to the validation of the active ingredient in the study of the spectrum–effect relationships.

### 4.2. Mechanism Exploration Based on Network Pharmacology and Molecular Docking

Network pharmacology based on the “multi-component, multi-target, multi-pathway” paradigm is a new pattern of drug research to analyze the relationship between drugs, targets, and metabolic pathways by building a network model [110]. The combination of network pharmacology and spectrum–effect relationship studies can establish drug–target–pathway networks of active ingredients and predict the effects of these active ingredients on some key targets and their pathways to explore the pharmacodynamic mechanism. For example, to explore the pharmacodynamic material basis of the *Curcuma wenyujin Y. H. Chen et C. Ling* rhizome for removing blood stasis, the target sites and metabolic pathways of the 10 components in plasma were predicted by network pharmacology [111]. The networks predicted that 80 targets were closely related to 10 components, among which 48 targets were related to 159 metabolic pathways such as arachidonic acid metabolism, sphingolipid signaling pathway and linoleic acid metabolism. However, although network pharmacology illustrates the association between “component–target–pathway”, it mostly lacks experimental correlation verification.

Molecular docking is a method of screening compounds based on the characteristics of the receptor target protein and the way in which the receptor target protein and the compound interact, and it can also be used to reverse screen the receptor that binds to the compound [112]. It is a theoretical simulation of interactions between molecules, such as ligands and receptors, and it predicts their binding patterns and affinity [113]. In the study of the spectrum–effect relationships, molecular docking technology is often used to further narrow the range of active compounds and reveal the possible mechanism of active compounds from the level of the structure–activity relationship. Through the analysis of the spectrum–effect relationships combined with the molecular docking strategy, Zhu et al.’s results showed that quercetin 3-*O*-rhamnosid-7-*O*-glucoside from *Sceptridium ternatum* binding to interleukin-6 has a high anti-inflammatory activity [114]. This strategy has also been successfully applied to the molecular docking of antiurolithic activity components in *Glechomae Herba* with CaSR receptors [115] and the antidiabetic components in Uncariae Rammulus Cum Uncis with α-glucosidase [116].

### 4.3. Pharmacodynamic Prediction Based on Chemometric Methods

The spectrum–effect relationships have the characteristic of associating the fingerprints of TCM with the actual efficacy, which can be used to predict the efficacy of TCM. By means of stoichiometric methods, a spectral effect relationship model, which is associated with the peak information and the efficacy information of multiple samples, can be trained. Through this model, the corresponding efficacy can be predicted by using the known sample peak information. At present, it has been successfully used to predict the efficacy of antioxidant [27], hemostatic [117], and immune activity [118]. However, applying this strategy requires strict control of the number of samples in the model. A small sample size and less input information will lead to a lack of robustness of the model and the accuracy of the prediction.

### 4.4. Evaluation of the Quality of Multi-Components Based on QAMS

In 2006, Wang et al. proposed for the first time that the QAMS method was used to evaluate the quality of multi-components in TCM, which solved the problem of limiting the quantitative analysis of multi-components due to the lack of reference materials [119]. In multi-index quantitative analysis, the QAMS method takes a certain component (with a reference supplier) as the internal standard, establishes a relative correction factor (RCF) between this component and other components, and calculates the content of other components through the RCF. Compared with the current multi-index component quantitative methods, QAMS has the characteristics of economy and accuracy, so it has been widely popularized and applied. By combining the QAMS method with the study of spectrum–effect relationships, the active components of medicinal materials can be determined through spectrum–effect relationships, and the chemical components found can be comprehensively evaluated by the QAMS method [120]. For examples, scopoletin was used as the internal standard to establish a QAMS quality control evaluation model for *Porana sinensis* Hemsl. [121], and chlorogenic acid was used as the internal standard to complete a comprehensive determination of the four main components of the hemostatic activity of *Blumea riparia* [122].

## 5. Conclusions and Future Perspectives

As an effective approach for quality control of TCM, the research of spectrum–effect relationships relates the fingerprints with the pharmacodynamic effect through chemometric methods, explores the biologically active components that play a curative role in TCM, and reflects the pharmacodynamic and intrinsic quality more comprehensively and accurately. After nearly 20 years of exploration, some interesting results have been obtained, such as the quality evaluation, pharmaceutical process optimization, and the exploration of drug-containing serum of TCM. Moreover, some novel strategies have also been applied to the research of spectrum–effect relationships, such as efficacy verification by monomeric compound and component knock-out, mechanism exploration based on network pharmacology and molecular docking, pharmacodynamic prediction based on chemometric methods, and evaluating the quality of multi-components based on QAMS. Despite 20 years of continuous updates and development, after this comprehensive review, it is not difficult to find that there is still room for progress in the research of spectrum–effect relationships.

The smallest component unit of both formulas and commercialized compound preparations is a single herb, which may be the reason why most of the current research objects focus on single herbs. However, in the subsequent research of spectrum–effect relationships, the interaction between different TCMs in the compound and preparation, such as “Jun-Chen-Zuo-Shi”, should be solved as a major research problem. Because in the current clinical treatment process, a single Chinese medicine is rarely used to treat diseases. Similarly, due to the low bioavailability of some components, studies of drug-containing serums should be of interest as a possible way to bring researchers closer to the real bioactive compounds. However, such experiments tend to be complex and require more resources.

The chemical composition of natural products with complex substrates is extremely complex. Although many components have a low content in the fingerprints, they play a decisive role in the pharmacodynamic activity of TCM, which is often ignored by researchers. Therefore, how to accurately select the chemical components in the fingerprints is a problem to be solved. At present, H/UHPLC is the most commonly used method to establish fingerprints in the research of spectrum–effect relationships. Due to the requirements of detection limit, separation degree and compound identification, H/UHPLC combined with an MS detector could realize the structural identification of active unknown compounds. In addition, compounds of different polarity, molecular weight and functional groups can be detected by different analytical methods to obtain comprehensive chemical profile information.

Furthermore, although TLC is not used much in the research of spectrum–effect relationships, it has a non-negligible advantage in fingerprint analysis. With the development of novel chromatographic plates in recent years [123], more automated instruments [124], advanced image analysis techniques, and even smartphones [125,126], more precise and rapid quantification of spots can be performed. TLC can also be used in combination with other technologies, such as fingerprints for screening active constituents with antioxidant activity [127] and direct analysis of bioactive compounds in natural products with laser ablation-assisted direct analysis in real-time mass spectrometry [128]. With the improvement of TLC, this technology will be more suitable for researching spectrum–effect relationships.

At present, more molecular and cellular models are used in pharmacodynamic studies, which provides great convenience for the high-throughput screening of drug efficacy in the early stage. However, it is still necessary to note that TCM, which relies on a holistic view to reflect the efficacy, should take into account a more ideal in vivo model to ensure the accuracy and applicability of the research results of spectrum–effect relationships. Objectively, there are also some tricky problems with in vivo modeling. In the clinic, Chinese medicine pays attention to dialectical treatment. Different syndromes and different body states may lead to different considerations in TCM’s treatment of diseases. As a result, it is difficult to fully replicate a representative model of the disease. In addition, there are few reports on the use of modern pharmacodynamic evaluation methods based on traditional efficacy in the current research, which suggests that the study of spectrum–effect relationships not only explores the basis of pharmacodynamic substances but also has further development prospects in the study of the potential material basis of the traditional properties of TCM, such as “medicinal property” and “medicinal flavor”. In addition, in the evaluation of pharmacological indicators, some software programs (such as Molinspiration, Swiss TargetPrediction, SuperPred, or others) can also be used to screen the activity of compounds and predict the target so that the research of spectrum–effect relationships could be more efficient and in-depth.

Multivariate statistical methods can be used to establish the spectrum–effect relationships of TCM through mathematical models. However, only a few studies have verified the predicted results using monomer compounds, and further verification of the results is needed. Regarding statistical analysis, most studies use GRA and PLS to establish the spectrum–effect relationships model, and there is no uniform regulation on which data analysis method to use for analysis. Most subsequent researchers imitate the previous researchers and adopt the same data analysis method. At the same time, most current studies only use one data analysis method to establish the spectrum–effect relationships model. The modeling results through a single method may have limitations. Subsequent research should try to combine a variety of statistical analysis methods to explore the effective information in the model from multiple perspectives.

The research of spectrum–effect relationships has been successfully applied in the quality evaluation, pharmaceutical process optimization, and exploration of drug-containing serum of TCM. The study of spectrum–effect relationships should not only be limited to the methods and technologies established at present but also need to break through the bottleneck of online analysis technology and online activity detection. For example, the combination of online antioxidant activity determination and spectrum–effect relationships could complete the simultaneous fingerprints and efficacy detection and realize the high-throughput online determination of active compounds, thus providing a new idea for the innovative development of the spectrum–effect relationships.

In short, with the rapid development of analytical instruments and analytical techniques, the continuous maturity of pharmacodynamic models and pharmacodynamic indexes that fit the overall view and the gradual standardization and diversification of data processing methods, the spectrum–effect relationships, as an effective means of quality control for TCM, will be able to provide a more comprehensive reference for the standards of quality control and process optimization of TCM.

## Figures and Tables

**Table 1:** Comparison of different analytical methods in the study of the spectrum–efficiency relationship.

Method	Advantage	Limitation
H/UHPLC-UV/DAD	It is suitable for most compounds, and has the advantages of simple operation, strong specificity, good repeatability, high sensitivity, wide linear range and low cost	Long analysis time, large solvent consumption; compounds without UV–visible light absorption cannot be detected
H/UHPLC-ELSD	A universal detector that can detect a variety of samples with lower volatility than the mobile phase	Low sensitivity, especially for compounds with UV absorption; high requirements for mobile phase
H/UHPLC-MS	High throughput detection, structure analysis of unknown compounds, high stability, sensitivity and repeatability	Expensive equipment, easy contamination of ion sources, and complex operation
GC-FID	Destructive mass type universal detector with wide linear range, high separation efficiency, fast analysis speed, small sample dosage and high detection sensitivity	Long analysis time, a known compound is required for qualitative analysis
GC-MS	High qualitative reliability, high sensitivity, suitable for most volatile compounds	Expensive equipment, not suitable for analysis of thermally unstable compounds
CE-UV	Short analysis time, less solvent consumption, less sample demand, low cost; and the sample does not require color development or dyeing with fluorescein dyes	Only compounds containing aromatic molecules or containing conjugated structures absorbed in the UV range can be detected
UV	Non-destructive analysis, fast analysis, low cost of use	Poor repeatability, serious spectral overlap and poor accuracy
IR	Fast analysis, does not consume solvents, no sample preparation, low cost	Low accuracy in quantification, low specificity
TLC	Simple operation, low equipment requirements, fast analysis speed	Quantitative difficulty and poor separation of polymer compounds
ICP-MS	Suitable for simultaneous detection of multiple elements and low detection limit	Expensive system, low automation, and serious signal drift
Combination methods	The chemical composition information reflected by different instrumental analysis methods or detection conditions is more comprehensive	-

**Table 2:** Comparison of the chemometric methods applied in spectrum–effect relationships.

Method	Characteristic	Advantage	Disadvantage
GRA	By measuring the degree of similarity or difference in the development trend of variables, the correlation between variables is measured	Can use the known information to reveal unknown information, less data demand, low data requirements	Difficult to describe the overall contribution of the corresponding components of peaks
BCA	The degree of correlation between the two variables was analyzed using the original data of the sample	Reflect the direction between two variables	Integrality is ignored, and the synergistic effect of various peaks on pharmacodynamics cannot be explained
HCA	An unsupervised analysis method in which samples or variables are categorized according to their degree of similarity	Intuitive, concise and achieve the preliminary analysis before actual modeling	Cannot reflect the magnitude and direction of correlation between fingerprint peaks and pharmacodynamic indexes
ANNs	Consider the complexity of the internal function of the existing information and the ambiguity of the relationship	Nonlinear fitting ability, simplify the data and self-adaptation	Long model training time, slow convergence speed and unstable memory system, overfitting may occur for small sample data, black box model, the mechanism is hard to understand
MLR	Establish a linear relationship between a dependent variable and the independent variable	Predict and analyze the difficult index through the easy index	Not enough to ensure the accuracy of the model when the relationship between independent variables is a multiple linear relationship
PLSR	Establish a linear relationship between dependent and independent variables in the case of multiple variables	Less data demand, maximum use of data information, small computation, and high prediction accuracy	Results are difficult to interpret due to the definition of latent variables
CCA	Reflect the overall correlation between the two groups of indicators by using the correlation between the two canonical variables	Simplify complex data, quantitatively describe the degree of linear correlation	Dimensionality reduction reduces the data content; only the correlation between one variable and one variable is considered, and the correlation between variables within the variable group cannot be considered.
PCA	Reduce the dimension of data while maintaining the maximum contribution to the variance of data	Extracting data, removing redundant information	The meaning of the principal component characteristic dimension is fuzzy and poor in interpretation; dimensionality reduction may result in data loss

**Table 5:** Application of spectrum–effect relationships in exploring the drug-containing serum of TCM.

TCM	Fingerprints	Pharmacodynamic	Animal	Chemometrics	Component	Reference
Black ginseng	HPLC	Anti-cancer activity (MTT assay of prostate cancer cell-DU145)	Rats	BCA and GRA	The active components of Black ginseng against prostate cancer were mainly ginsenosides Rg5, S-Rg3, R-Rg3, RK1 and S-Rg2.	[102]
Yangyin Tongnao Granules	HPLC	Inflammatory factors (tumor necrosis factor-α and interleukin-18)	Cerebral ischemia-reperfusion injury rats	GRA, MLR, and PLSR	Six components	[103]
Fangji Huangqi Tang	UHPLC-ESI-Q-TOF-MS	Anti-adriamycin nephrosis biological effect (cystatin C, blood urea nitrogen and serum creatinine)	Rats	CCA	Tetrandrine, N-methylfangchinoline, tetrandrine-M2, tetrandrine-M3, tetrandrine-M4, fangchinoline, curine, licoricone-M1, licochalcone B-M3, fenfangjine F-M2 and glycyrrhetic acid	[21]
Dahuang Zhechong Pill	UPLC	Anti-hepatoma effect (HepG2 and HUVEC cells)	Liver cirrhosis and hepatocellular carcinoma rats induced by diethylnitrosamine	PLSR	Allantoin, hypoxanthine, salidroside, hydroxypaeoniflorin, liquiritin, isoliquiritin and other 26 components	[104]
Shuang Huang Lian Injection	UPLC	Screening anaphylactoid components (RBL-2H_3_ cells)	Rats	Regression analysis and CCA	Chlorogenic, syringin, beferulic acid, 5-hydroxy-7,3’,4’,5’-7-tetramethoxy flavone, caffeic acid methyl ester	[105]

## Data Availability

Not applicable.
